# Supplementary material for: A FYVE zinc finger domain protein specifically links mRNA transport to endosome trafficking
Source: eLife. 2015 May 18;4:e06041. doi: 10.7554/eLife.06041 (PMC4466420; doi:10.7554/eLife.06041)
Supplement: Supplementary file 1. — Potential PAM2-containing proteins from U. maydis. DOI: http://dx.doi.org/10.7554/eLife.06041.047 [file elife06041s001.rtf]

Supplementary file 1: Potential PAM2-containing proteins from U. maydis
Upa	UMAG	Length [aa]	Put. PAM2 motifs	Other domains 
[SMART1]	Homologues in S. cerevisisae 
[% aa  identity]	
1	12183	1287	1	FYVE, RING, Ankyrin repeats	Pib1 [30]	
2	10350	2121	4	no known domains	none	
3	02637	1049	1	Sm, LsmAD	Pbp1 [28]	
4	03521	633	1	no known domains	none	
5	00427	1187	1	no known domains	none	
6	00859	379	1	THOC7	none	
7	04609	1092	1	ZnFinger_CCCH-type, DUF1771, Smr	none	
8	05695	755	1	GTP_EfTUI-III	eRF3 [57]	
9	06200	585	1	no known domains	Pex3 [23]	
10	03505	1945	1	SAC3_GANP	Sac3 [33]	
11	01657	1928	1	no known domains	none	
12	00883	1633	2	IPK	Kcs1 [45]	
13	02501	866	1	ZnFinger_CCCH-type, S_TKc	Pan3 [33]	
14	05540	2118	1	Not1, DUF3819	Not1 [27]	
1 http://smart.embl-heidelberg.de/
