# Supplementary material for: A FYVE zinc finger domain protein specifically links mRNA transport to endosome trafficking
Source: eLife. 2015 May 18;4:e06041. doi: 10.7554/eLife.06041 (PMC4466420; doi:10.7554/eLife.06041)
Supplement: Supplementary file 2. — Description of U. maydis strains used in this study. DOI: http://dx.doi.org/10.7554/eLife.06041.048 [file elife06041s002.rtf]

Supplementary file 2: Description of U. maydis strains used in this study
Strain	Locus	Progenitor strain	Short description	
AB33	b	FB2	Pnar:bW2bE1, expression of active b heterodimer under control 
of the nar1 promoter, strain grows filamentously upon changing the 
nitrogen source._ENREF_1	
AB33rrm4D	rrm4	AB33	carrying a deletion of rrm4.	
AB33upa1D	upa1	AB33	carrying a deletion of upa1.	
AB33upa1-Gfp	upa1	AB33	expressing Upa1 C-terminally fused to eGfp.	
AB33upa1mP-Gfp	upa1	AB33upa1D	expressing Upa1mP C-terminally fused to eGfp. Upa1mP carries the amino acid substitutions L132A, A136S, F139A and P141A in the PAM2-motif.	
AB33upa1DN1-Gfp	upa1	AB33upa1D	expressing Upa1DN1 C-terminally fused to eGfp. Like Upa1-Gfp, but carrying a N-terminal truncation from aa 1-143 including the PAM2-motif.	
AB33upa1DN2-Gfp	upa1	AB33upa1D	expressing Upa1DN2 C-terminally fused to eGfp. Like Upa1-Gfp, but carrying a N-terminal truncation from aa 1-337 including the PAM2 and PAM2L-motifs.	
AB33upa1DN3-Gfp	upa1	AB33upa1D	expressing Upa1DN3 C-terminally fused to eGfp. Like Upa1-Gfp, but carrying a N-terminal truncation from aa 1-551.	
AB33upa1DN4-Gfp	upa1	AB33upa1D	expressing Upa1DN4 C-terminally fused to eGfp. Like Upa1-Gfp, but carrying a N-terminal truncation from aa 1-633.	
AB33upa1DN5-Gfp	upa1	AB33upa1D	expressing Upa1DN5 C-terminally fused to eGfp. Like Upa1G, but carrying a N-terminal truncation from aa 1-719.	
AB33upa1DN6-Gfp	upa1	AB33upa1D	expressing Upa1DN6 C-terminally fused to eGfp. Like Upa1-Gfp, but carrying a N-terminal truncation from aa 1-969.	
AB33upa1-Gfp/kin3D	upa1
kin3	AB33upa1-Gfp	expressing Upa1 C-terminally fused to eGfp and carrying a deletion of kin3.	
AB33upa1DR-Gfp	upa1	AB33	expressing Upa1DR C-terminally fused to eGfp. Like Upa1-Gfp, but carrying a C-terminal truncation from aa 1241-1287 including the RING domain.	
AB33upa1DFR-Gfp	upa1	AB33	expressing Upa1DFR C-terminally fused to eGfp. Like Upa1-Gfp, but carrying a C-terminal truncation from aa 1048-1287 including the FYVE domain as well as the RING domain.	
AB33upa1-Gfp/rab5a-Cherry	upa1
ipS	AB33upa1-Gfp	expressing Upa1 C-terminally fused to eGfp and Rab5a N-terminally fused to mCherry. Rab5a-Cherry is ectopically integrated in the defined ipS locus and under control of the constitutive Ptef promoter.	
AB33rrm4-Rfp/upa1-Gfp	rrm4
upa1	AB33rrm4-Rfp	expressing Rrm4 C-terminally fused to Rfp and Upa1 C-terminally fused to eGfp.	
AB33Potefrab5a-Gfp	rab5a	AB33	expressing Rab5a N-terminally fused to eGfp. Rab5a-Gfp is ectopically integrated in the defined ipS locus and under control of the strong constitutive Potef promoter.	
AB33Potefrab5a-Gfp/upa1D	rab5a
upa1	AB33Potefrab5a-Gfp	carrying a deletion of upa1 and expressing Rab5a N-terminally fused to eGfp. Rab5a-Gfp is ectopically integrated in the defined ipS locus and under control of the strong constitutive Potef promoter.	
AB33Ptefrab5a-Gfp	rab5a	AB33	expressing Rab5a N-terminally fused to eGfp. Rab5a-Gfp is ectopically integrated in the defined ipS locus and under control of the constitutive Ptef promoter.	
AB33Ptefrab5a-Gfp/upa1D	rab5a
upa1	AB33Ptefrab5a-Gfp	carrying a deletion of upa1 and expressing Rab5a N-terminally fused to eGfp. Rab5a-Gfp is ectopically integrated in the defined ipS locus and under control of the constitutive Ptef promoter.	
AB33rrm4-Gfp	rrm4	AB33rrm4D	expressing Rrm4 C-terminally fused to eGfp.	
AB33rrm4-Gfp/upa1D	rrm4
upa1	AB33rrm4-Gfp	expressing Rrm4 C-terminally fused to eGfp and carrying a deletion of upa1.	
AB33pab1-Gfp	pab1	AB33	expressing Pab1 C-terminally fused to eGfp.	
AB33pab1-Gfp/upa1D	pab1
upa1	AB33pab1-Gfp	expressing Pab1 C-terminally fused to eGfp and carrying a deletion of upa1.	
AB33rrm4-Cherry/rps2-Gfp	rrm4
rps2	AB33rrm4-Cherry	expressing Rrm4 C-terminally fused to mCherry and Rps2 C-terminally fused to Gfp. rps2-Gfp is ectopically integrated in the defined ipS locus and under control of the constitutive Potef promoter. 	
AB33rrm4-Cherry/rps2-Gfp/upa1D	rrm4
rps2
upa1	AB33rrm4-Cherry /rps2-Gfp	expressing Rrm4 C-terminally fused to mCherry and Rps2 C-terminally fused to Gfp. rps2-Gfp is ectopically integrated in the defined ipS locus and under control of the constitutive Potef promoter. Additionally carrying a deletion of upa1.	
AB33rps2-Gfp/rrm4D	rrm4
rps2	AB33rrm4-Cherry /rps2-Gfp	carrying a deletion of rrm4 and expressing Rps2 C-terminally fused to Gfp. rps2-Gfp is ectopically integrated in the defined ipS locus and under control of the constitutive Potef promoter. 	
AB33rrm4-Cherry/rpl25-Gfp	rrm4
rpl25	AB33rrm4-Cherry	expressing Rrm4 C-terminally fused to mCherry and Rpl25 C-terminally fused to Gfp. rpl25-Gfp is ectopically integrated in the defined ipS locus and under control of the constitutive Potef promoter. 	
AB33rrm4-Cherry/rpl25-Gfp/upa1D	rrm4
rps25
upa1	AB33rrm4-Cherry /rps2-Gfp	expressing Rrm4 C-terminally fused to mCherry and Rpl25 C-terminally fused to Gfp. rpl25-Gfp is ectopically integrated in the defined ipS locus and under control of the constitutive Potef promoter. Additionally carrying a deletion of upa1.	
AB33rrm4-Cherry/rps19-Gfp	rrm4
rps19	AB33rrm4-Cherry	expressing Rrm4 C-terminally fused to mCherry and Rps19 C-terminally fused to Gfp.	
AB33rrm4-Cherry/rps19-Gfp /upa1D	rrm4
rps19
upa1	AB33rrm4-Cherry /rps19-Gfp	expressing Rrm4 C-terminally fused to mCherry and Rps19 C-terminally fused to Gfp. Additionally carrying a deletion of upa1.	
AB33cdc3B16/lN*NLS-Gfp3	cdc3
ipS	AB33cdc3B16	expressing lN*-peptide fused to triple eGfp and cdc3 mRNA tagged with 16 copies of boxB binding site.	
AB33cdc3B16/lN*NLS-Gfp3 /rrm4-Cherry	cdc3
ipS
rrm4	AB33cdc3B16/
lN*NLS-Gfp3	expressing Rrm4 C-terminally fused to mCherry and lN*-peptide fused to triple eGfp and cdc3 mRNA tagged with 16 copies of boxB binding site.	
AB33cdc3B16/lN*NLS-Gfp3 /upa1D	cdc3
ipS
upa1	AB33cdc3B16/
lN*NLS-Gfp3	expressing and lN*-peptide fused to triple eGfp and cdc3 mRNA tagged with 16 copies of boxB binding site. Additionally carrying a deletion of upa1.	
AB33cdc3-Gfp	cdc3	AB33	expressing Cdc3 N-terminally fused to eGfp.	
AB33cdc3-Gfp/upa1D	cdc3
upa1	AB33cdc3-Gfp	expressing Cdc3 N-terminally fused to eGfp and carrying a deletion of upa1.	
AB33rrm4-Cherry/upa1mPL-1-Gfp	rrm4
upa1	AB33rrm4-Cherry/ upa1D	expressing Rrm4 C-terminally fused to mCherry and Upa1mPL-1 C-terminally fused to eGfp. Upa1mPL-1 carries block mutations leading to the amino acid substitutions AASAAATAAS from aa 242-251 in the N-terminal PAM2L-motif (PAM2L-1).	
AB33rrm4-Cherry/upa1mPL-2-Gfp	rrm4
upa1	AB33rrm4-Cherry/ upa1D	expressing Rrm4 C-terminally fused to mCherry and Upa1mPL-2 C-terminally fused to eGfp. Upa1mPL-2 carries block mutations leading to the amino acid substitutions AASAAATAAS from aa 949-958 in the C-terminal PAM2L-motiv (PAM2L-2).	
AB33rrm4-Cherry/upa1mPL-1+2-Gfp	rrm4
upa1	AB33rrm4-Cherry/ upa1D	expressing Rrm4 C-terminally fused to mCherry and Upa1mPL-1 C-terminally fused to eGfp. Upa1mPLN-2 carries block mutations leading to the amino acid substitutions AASAAATAAS from aa 242-251 in the N-terminal PAM2L-motif (PAM2L-1) and from aa 949-958 in the C-terminal PAM2L-motiv (PAM2L-2).	
AB33rrm4-Cherry/ upa1mP/PL-1+2-Gfp	rrm4
upa1	AB33rrm4-Cherry/ upa1D	Upa1mP C-terminally fused to eGfp. Upa1mP carries the amino acid substitutions L132A, A136S, F139A and P141A in the PAM2-motif, as well as the block mutations leading to the amino acid substitutions AASAAATAAS from aa 242-251 in the N-terminal PAM2L-motif (PAM2L-1) and from aa 949-958 in the C-terminal PAM2L-motiv (PAM2L-2).	
AB33rrm4-Gfp/rab5a-Cherry	rrm4
rab5a	AB33rrm4-Gfp	expressing Rrm4 C-terminally fused to eGfp and Rab5a N-terminally fused to mCherry. Rab5a-Cherry is ectopically integrated in the defined ipS locus and under control of the constitutive Ptef promoter.	
AB33rrm4-Gfp-/rab5a-Cherry
/upa1D	rrm4
rab5a
upa1	AB33rrm4-Gfp/ upa1D	expressing Rrm4 C-terminally fused to eGfp and Rab5a N-terminally fused to mCherry. Rab5a-Cherry is ectopically integrated in the defined ipS locus and under control of the constitutive Ptef promoter. Additionally carrying a deletion of upa1.	
AB33rrm4-Gfp-TAP/yup1-Cherry	rrm4
yup1	AB33rrm4-Gfp-TAP	expressing Rrm4 C-terminally fused to eGfp followed by an Tandem affinity purification tag and Yup1 C-terminally fused to mCherry followed by a triple c-Myc-tag. Yup1-Cherry is ectopically integrated in the defined ipS locus and under control of the constitutive Ptef promoter.	
AB33rrm4-Gfp-TAP/yup1-Cherry/upa1D	rrm4
yup1
upa1	AB33rrm4-Gfp-TAP/yup1-Cherry	expressing Rrm4 C-terminally fused to eGfp followed by an Tandem affinity purification tag and Yup1 C-terminally fused to mCherry. Yup1-Cherry is ectopically integrated in the defined ipS locus and under control of the constitutive Ptef promoter.Additionally carrying a deletion of upa1.	
