# Supplementary material for: A FYVE zinc finger domain protein specifically links mRNA transport to endosome trafficking
Source: eLife. 2015 May 18;4:e06041. doi: 10.7554/eLife.06041 (PMC4466420; doi:10.7554/eLife.06041)
Supplement: Supplementary file 3. — Generation of U. maydis strains used in this study. DOI: http://dx.doi.org/10.7554/eLife.06041.049 [file elife06041s003.rtf]

Supplementary file 3: Generation of U. maydis strains used in this study
Strains	Relevant genotype	Uma	Reference	Transformed
plasmid	Locus	Progenitor	
AB33
	a2 Pnar:bW2 bE1	133	Brachmann, 2001	pAB33	b	FB2	
AB33rrm4D	rrm4D
	273	Becht, 2006	pRrm4D_HygR
(pUMa495)	rrm4
	AB33
	
AB33upa1D	upa1D	859	this study	pUpa1D_HygR
(pUMa1574)	upa1	AB33
	
AB33upa1-Gfp	upa1-Gfp 
	956	this study	pUpa1-Gfp_NatR
(pUMa1575)	upa1	AB33
	
AB33upa1mP-Gfp	upa1mP-Gfp	1016	this study	pUpa1mP-Gfp _NatR
(pUMa1786)	upa1	AB33upa1D	
AB33upa1DN1-Gfp	upa1DN1-Gfp	1198	this study	pUpa1 N1D-Gfp _NatR
(pUMa1969)	upa1	AB33upa1D	
AB33upa1DN2-Gfp
	upa1DN2-Gfp
	1199
	this study
	pUpa1N2D -Gfp _NatR
(pUMa1970) 	upa1
	AB33upa1D
	
AB33upa1DN3-Gfp
	upa1DN3-Gfp
	1241	this study	pUpa1N3D-Gfp _NatR (pUMa2051)	upa1	AB33upa1D	
AB33upa1DN4-Gfp
	upa1DN4-Gfp
	1242	this study	pUpa1N4D -Gfp _NatR (pUMa2052)	upa1	AB33upa1D	
AB33upa1DN5-Gfp	upa1DN5-Gfp	1243	this study	pUpa1N5D -Gfp _NatR (pUMa2053)	upa1	AB33upa1D	
AB33upa1DN6-Gfp
	upa1DN6-Gfp
	1244
	this study
	pUpa1N6D -Gfp _NatR (pUMa2029)	upa1
	AB33upa1D	
AB33upa1-Gfp/kin3D
	upa1-Gfp
kin3D	1163
	this study
	pKin3D-GenitR (pUMa1288)	kin3	AB33upa1-Gfp	
AB33upa1DR-Gfp
	upa1DR-Gfp	1059	this study	pUpa1DR-Gfp _NatR
(pUMa1739)	upa1	AB33
	
AB33upa1DFR-Gfp	upa1DFR-Gfp	1061	this study	pUpa1DFR-Gfp _NatR
(pUMa1740)	upa1	AB33	
AB33upa1-Gfp/rab5a-Cherry	upa1-Gfp
rab5a-Cherry	1055	this study	pPtefRab5a-Cherry _CbxR (pUMa1806)	ipS	AB33upa1-Gfp
	
AB33rrm4-Rfp/upa1-Gfp	upa1-Gfp 
rrm4-Rfp	957	this study	pUpa1-Gfp_NatR (pUMa1575)	upa1	AB33rrm4-Rfp
	
AB33Potefrab5a-Gfp	rab5a-Gfp	826	this study	pPotefRab5a-Gfp _CbxR (pUMa1481)	rab5a	AB33	
AB33Potefrab5a-Gfp/upa1D	rab5a-Gfp
upa1D	1252	this study	pUpa1D_GenitR
(pUMa1915)	upa1	AB33rab5a-Gfp	
AB33Ptefrab5a-Gfp	rab5a-Gfp	988	this study	pPtefRab5a-Gfp _CbxR (pUMa1712)	rab5a	AB33	
AB33Ptefrab5a-Gfp/upa1D	rab5a-Gfp
upa1D	1344	this study	pUpa1D_GenitR
(pUMa1915)	upa1	AB33rab5a-Gfp	
AB33rrm4-Gfp	rrm4-Gfp	274	Becht, 2006	pRrm4-Gfp_NatR
(pUMa496)	rrm4	AB33rrm4D	
AB33rrm4-Gfp/upa1D	rrm4-Gfp
upa1D	856	this study	pUpa1D_HygR
(pUMa1574)	upa1	AB33rrm4-Gfp 	
AB33pab1-Gfp	pab1-Gfp 
	389	König, 2009	pPab1-Gfp_NatR 
(pUMa805)	pab1	AB33
	
AB33pab1-Gfp/upa1D	pab1-Gfp
upa1D	857	this study	pUpa1D_HygR
(pUMa1574)	upa1	AB33pab1-Gfp	
AB33rrm4-Cherry/rps2-Gfp	rrm4-Chrrye
rps2-Gfp	1003	Baumann, 2014	pPotefRps2-Gfp
(pUMa1725)	ipS	AB33rrm4-Cherry	
AB33rrm4-Cherry/rps2-Gfp /upa1D	rrm4-Cherry
rps2-Gfp
upa1D	1390	this study	pUpa1D_GenitR
(pUMa1915)	upa1	AB33rrm4-Cherry
/rps2-Gfp	
AB33rps2-Gfp/rrm4D	rps2-Gfp
rrm4D	1008	Baumann, 2014 	pRrm4D_HygR
(pUMa865)	rrm4	AB33rrm4-Cherry
/rps2-Gfp 	
AB33rrm4-Cherry/rpl25-Gfp	rrm4-Cherry
rpl25-Gfp	993	Baumann, 2014	pPotefRpl25-Gfp
(pUMa1726)	ipS	AB33rrm4-Cherry	
AB33rrm4-Cherry/rpl25-Gfp /upa1D	rrm4-Cherry
rpl25-Gfp
upa1D	1274	this study	pUpa1D_GenitR
(pUMa1915)	upa1	AB33rrm4-Cherry
/rpl25-Gfp	
AB33rrm4-Cherry/rps19-Gfp	rrm4-Cherry
rps19-Gfp	1262	this study	pRps19-Gfp_NatR
(pUMa829)	Rps19	AB33rrm4-Cherry	
AB33rrm4-Cherry/rps19-Gfp/upa1D	rrm4-Cherry
rps19-Gfp
upa1D	1273	this study	pUpa1D_GenitR
(pUMa1915)	upa1	AB33rrm4-Cherry
/rps19-Gfp	
AB33cdc3B16/lN*NLS-Gfp3	cdc3B16
lN*NLS-Gfp3	874	this study	pPoteflN*3NLS-Gfp
_CbxR (pUMa1572)	ipS	AB33cdc3B16	
AB33cdc3B16/lN*NLS-Gfp3 /rrm4-Cherry	cdc3B16
lN*NLS-Gfp3 rrm4-Cherry	951	this study	pRrm4-Cherry
_HygR
(pUMa1468)	rrm4	AB33cdc3B16
/lN*NLS-Gfp3	
AB33cdc3B16/lN*NLS-Gfp3 /upa1D	cdc3B16
lN*NLS-Gfp3 upa1D	1372	this study	pUpa1D_GenitR
(pUMa1915)	upa1	AB33cdc3B16
/lN*NLS-Gfp3	
AB33cdc3-Gfp	cdc3-Gfp	449	Baumann, 2014	pCdc3-GfpN_NatR
(pUMa1028)	cdc3	AB33	
AB33cdc3-Gfp/upa1D	cdc3-Gfp
upa1D	1270	this study	pUpa1D_GenitR
(pUMa1915)	upa1	AB33cdc3-Gfp	
AB33rrm4-Cherry/upa1mPL-1-Gfp	rrm4-Cherry
upa1mPL-1-Gfp	1628	this study	pUpa1mPL-1-Gfp
_NatR
(pUMa2302)	upa1	AB33rrm4-Cherry/ upa1D	
AB33rrm4-Cherry/upa1mPL-2-Gfp	rrm4-Cherry
upa1mPL-2-Gfp	1579	this study	pUpa1mPL-2-Gfp
_NatR (pUMa2303)	upa1	AB33rrm4-Cherry/ upa1D	
AB33rrm4-Cherry/upa1mPL-1+2-Gfp	rrm4-Cherry
upa1 mPL-1+2-Gfp	1630	this study	pUpa1mPL-1+2-Gfp
_NatR (pUMa2305)	upa1	AB33rrm4-Cherry/ upa1D	
AB33rrm4-Cherry/ upa1mP/PL-1+2-Gfp	rrm4-Cherry
upa1mP/PL-1+2-Gfp	1631	this study	pUpa1mP/mPL-1+2-Gfp
_NatR (pUMa2572)	upa1	AB33rrm4-Cherry/ upa1D	
AB33rrm4-Gfp/rab5a-Cherry	rrm4-Gfp
rab5a-Cherry	1053	this study	pPtefRab5a-Cherry _CbxR (pUMa1806)	ipS	AB33rrm4-Gfp	
AB33rrm4-Gfp-/rab5a-Cherry
/upa1D	rrm4-Gfp
rab5a-Cherry
upa1D	1056	this study	pPtefRab5a-Chrrye _CbxR (pUMa1806)	ipS	AB33rrm4-Gfp/ upa1D	
AB33rrm4-Gfp-TAP/yup1-Cherry	rrm4-Gfp-TT
yup1-Cherry	686	Baumann, 2012	pPotefYup1-CherryMyc_CbxR	ipS	AB33rrm4-Gfp-TAP	
AB33rrm4-Gfp-TAP/yup1-Cherry
/upa1D	rrm4-Gfp-TT
yup1-Cherry
upa1D	992	this study	pUpa1D_HygR
(pUMa1574)	upa1	AB33rrm4-Gfp-TAP
/yup1-Cherry	
