# Supplementary material for: A FYVE zinc finger domain protein specifically links mRNA transport to endosome trafficking
Source: eLife. 2015 May 18;4:e06041. doi: 10.7554/eLife.06041 (PMC4466420; doi:10.7554/eLife.06041)
Supplement: Supplementary file 4. — Description of plasmids used for U. maydis strain generation. DOI: http://dx.doi.org/10.7554/eLife.06041.050 [file elife06041s004.rtf]

Supplementary file 4: Description of plasmids used for U. maydis strain generation
Plasmid	pUMa	Resistance cassette	Short description	
pUpa1D_HygR	1574	SfiI-insert of MF1hs	Plasmid for generating deletion mutants of upa1. Resistance cassette is flanked by 1 kb upstream and 1.9 kb downstream region of upa1. Flanking regions were amplified by PCR using oRl305/oRL307 and oRL306/oRL309 and UM521 wild-type DNA as template. 	
pUpa1-Gfp_NatR	1575	SfiI-insert of pMF5-1n	Plasmid for generating eGfp fusions of upa1. A cassette containing Gfp, the Tnos terminator and a nourseothricin resistance cassette is flanked by 4.9 kb upstream region including 3.9 kb of the upa1 ORF and 1.9 kb downstream region of upa1. Flanking regions were amplified by PCR using oRl305/oRL308 and oRL306/oRL309 and UM521 wild-type DNA as template.	
pUpa1mP-Gfp_NatR	1786	SfiI-insert of pMF5-1n	Plasmid for generating eGfp fusions of upa1mP. Like pUpa1-Gfp_NatR, but carrying the nucleic acid exchanges T394G, T395C, G396C, G406C, C407T, T415G, T416C, G420A, C421G and G423T, resulting in the amino acid substitutions L132A, A136S, F139A and P141A in the PAM2-motif.	
pUpa1DN1-Gfp_NatR	1969	SfiI-insert of pMF5-1n	Plasmid for generating eGfp fusions of upa1DN1. Like pUpa1-Gfp_NatR, but carrying a N-terminal truncation from aa 1-143.	
pUpa1DN2-Gfp_NatR	1970	SfiI-insert of pMF5-1n	Plasmid for generating eGfp fusions of upa1DN2. Like pUpa1-Gfp_NatR, but carrying a N-terminal truncation from aa 1-357.	
pUpa1DN3-Gfp_NatR	2051	SfiI-insert of pMF5-1n	Plasmid for generating eGfp fusions of upa1DN3. Like pUpa1-Gfp_NatR, but carrying a N-terminal truncation from aa 1-551.	
pUpa1DN4-Gfp_NatR	2052	SfiI-insert of pMF5-1n	Plasmid for generating eGfp fusions of upa1DN4. Like pUpa1-Gfp_NatR, but carrying a N-terminal truncation from aa 1-633.	
pUpa1DN5-Gfp_NatR	2053	SfiI-insert of pMF5-1n	Plasmid for generating eGfp fusions of upa1DN5. Like pUpa1-Gfp_NatR, but carrying a N-terminal truncation from aa 1-719.	
pUpa1DN6-Gfp_NatR	2029	SfiI-insert of pMF5-1n	Plasmid for generating eGfp fusions of upa1DN6. Like pUpa1-Gfp_NatR, but carrying a N-terminal truncation from aa 1-969.	
pKin3D-GenitR	1288	SfiI-insert of pMF1g	Published (Baumann, 2012).	
pUpa1DR-Gfp_NatR	1739	SfiI-insert of pMF5-1n	Plasmid for generating eGfp fusions of upa1DR. Like pUpa1-Gfp_NatR, but carrying a C-terminal truncation from aa 1241-1287 including the RING domain	
pUpa1DFR-Gfp_NatR	1740	SfiI-insert of pMF5-1n	Plasmid for generating eGfp fusions of upa1DFR. Like pUpa1-Gfp_NatR, but carrying a C-terminal truncation from aa 1048-1287 including the FYVE domain as well as the RING domain.	
pPtefRab5a-Cherry
_CbxR	1806	CbxR for integration at ipS  locus	Plasmid for ectopical integration and expression of rab5a-Cherry. Contains the 911 bp ORF of rab5a N-terminally fused with mCherry flanked upstream and downstream by the constitutively active promoter Ptef and the transcriptional terminator Tnos upstream and downstream, respectively. 	
pPotefRab5a-Gfp
_CbxR	1481	CbxR for integration at ipS  locus	Plasmid for ectopical integration and expression of rab5a-Gfp. Contains the 911 bp ORF of rab5a N-terminally fused with eGfp flanked upstream and downstream by the strong constitutively active promoter Potef and the transcriptional terminator Tnos upstream and downstream, respectively.	
pPtefRab5a-Gfp_CbxR	1712	CbxR for integration at ipS  locus	Plasmid for ectopical integration and expression of rab5a-Gfp. Contains the 911 bp ORF of rab5a N-terminally fused with eGfp flanked upstream and downstream by the constitutively active promoter Ptef and the transcriptional terminator Tnos upstream and downstream, respectively.	
pUpa1D_GenitR	1915	SfiI-insert of pMF1g	Plasmid for generating deletion mutants of upa1. Like pUpa1D_HygR, but the resistance cassette was exchanged from HygR to GenitR using the SfiI cassette system (Brachmann, 2001).	
pPotef_lN*NLS –Gfp3
_CbxR	1597	CbxR for integration at ipS  locus	Plasmid for ectopical integration and expression of an artificial gene containing the CDS for the first 22 amino acids of the lN protein (Baumann et al., 2014) fused to triple Gfp and a nuclear localisation signal. The gene is under control of the promotor Potef.	
pRrm4-Cherry_HygR	1468	SfiI-insert of pMF5-5h	Published (Baumann, 2014).	
pYup1-CherryMyc
_CbxR	1376	CbxR for integration at ipS  locus	Published (Baumann, 2012)	
