# Supplementary material for: A FYVE zinc finger domain protein specifically links mRNA transport to endosome trafficking
Source: eLife. 2015 May 18;4:e06041. doi: 10.7554/eLife.06041 (PMC4466420; doi:10.7554/eLife.06041)
Supplement: Supplementary file 5. — Description of plasmids used for yeast two-hybrid analyses. DOI: http://dx.doi.org/10.7554/eLife.06041.051 [file elife06041s005.rtf]

Supplementary file 5: Description of plasmids used for yeast two-hybrid analyses
Plasmid	pUMa	Gene	Short description	
pGADT7-DS 	1624		Plasmid for the expression of hybrid proteins, N-terminally fused to a nuclear localisation signal (NLS) of the simian virus 40 (SV40), followed by the Gal4 activation domain (aa 768-881) and an HA-epitope for Western Blot detection. Resulting hybrid proteins are termed AD-“X”. For positive selection of transformants on minimal medium this plasmid carries a LEU2 auxotrophy marker. This plasmid contains two diverse SfiI-restriction sites for cloning purposes (Dualsystems Biotech, Schlieren, Switzerland).	
pGBKT7-SfiI MCS 	1625		Plasmid for the expression of hybrid proteins, N-terminally fused to the Gal4 DNA-binding domain (aa 1-147), followed by a c-Myc-epitope for Western Blot detection. Resulting hybrid proteins are termed BD-“X”. For positive selection of transformants on minimal medium, this plasmid carries a TRP1 auxotrophy marker. This plasmid contains two diverse SfiI-restriction sites for cloning purposes. (Clontech Laboratories, Inc., Mountain View, CA, USA).	
pGADT7-T 	1636		Plasmid for the expression of a N-terminal AD-fusion of the large T-antigen of SV40. It interacts with BD-p53 as a positive control (Clontech).	
pGBKT7-p53 	1638		Plasmid for the expression of a N-terminal BD-fusion of the murine p53. It interacts with AD-T as a positive control (Clontech).	
pGBKT7-Lam 	1637		Plasmid for the expression of a N-terminal BD-fusion with the human nuclear protein Lamin C, which shows no interaction with most proteins and serves as negative control (Clontech).	
pGADT7-Rrm4 	1629	rrm4	Plasmid for the expression of AD-Rrm4.	
pGBKT7-Pab1 	1632	pab1	Plasmid for the expression of BD-Pab1.	
pGADT7-Pab1 	1633	pab1	Plasmid for the expression of AD-Pab1.	
pGADT7-Pab1mM 	2008	pab1	Plasmid for the expression of BD-Pab1mM. Like BD-Pab1, but carrying the amino acid substitutions Y580A, V583A, K593A and I598A in the MLLE domain.	
pGADT7-MLLEPab1 	2009	pab1	Plasmid for the expression of BD- MLLEPab1. For this the last 86 aa of the pab1 ORF including the MLLE domain were fused to Gal4-BD and Myc.	
pGADT7-Upa1 	1731	upa1	Plasmid for the expression of AD-Upa1.	
pGADT7-Upa1DF 	1764	upa1	Plasmid for the expression of AD-Upa1DF. Like AD-Upa1, but a region from aa 814-1156 including the FYVE domain was removed.	
pGADT7-Upa1mPDF 	2044	upa1	Plasmid for the expression of AD- Upa1mPDF. Like AD-Upa1DF, but carrying the amino acid substitutions L132A, A136S, F139A and P141A in the PAM2-motif.	
pGADT7-Upa1DN1DF 	2057	upa1	Plasmid for the expression of AD- Upa1DN1DF. Like AD-Upa1DF, but carrying a N-terminal truncation from aa 1-143 including the PAM2-motif.	
pGADT7-Upa1DC1 	2002	upa1	Plasmid for the expression of AD-Upa1DC1. Like AD-Upa1, but carrying a C-terminal truncation from aa 195-1287.	
pGADT7-Rrm4DN1 	2182	rrm4	Plasmid for the expression of AD-Rrm4DN1. Like AD-Rrm4, but carrying a N-terminal truncation from aa 1-66.	
pGADT7-Rrm4DN2 	2183	rrm4	Plasmid for the expression of AD-Rrm4DN2. Like AD-Rrm4, but carrying a N-terminal truncation from aa 1-152 including the first RRM domain.	
pGADT7-Rrm4DN3 	2184	rrm4	Plasmid for the expression of AD-Rrm4DN3. Like AD-Rrm4, but carrying a N-terminal truncation from aa 1-258 including the first two RRM domains.	
pGADT7-Rrm4DN4 	2185	rrm4	Plasmid for the expression of AD-Rrm4DN4. Like AD-Rrm4, but carrying a N-terminal truncation from aa 1-419 including all three RRM domains.	
pGADT7-Rrm4DN5 	2352	rrm4	Plasmid for the expression of AD-Rrm4DN5. Like AD-Rrm4, but carrying a N-terminal truncation from aa 1-478.	
pGADT7-Rrm4DN6 	2353	rrm4	Plasmid for the expression of AD-Rrm4DN6. Like AD-Rrm4, but carrying a N-terminal truncation from aa 1-547.	
pGADT7-Rrm4DN7 	2354	rrm4	Plasmid for the expression of AD-Rrm4DN7. Like AD-Rrm4, but carrying a N-terminal truncation from aa 1-646 including the N-terminally situated MLLE domain.	
pGADT7-MLLERrm4 	2088	rrm4	Plasmid for the expression of AD- MLLERrm4. For this the last 84 aa of the rrm4 ORF including the MLLE domain were fused to Gal4-BD and Myc.	
pGADT7-Rrm4DMLLE 	1971	rrm4	Plasmid for the expression of AD-Rrm4DMLLE. Like AD-Rrm4, but carrying a C-terminal truncation from aa 709-792 including the C-terminally situated MLLE domain.	
pGADT7-Rrm4mMLLE 	1995	rrm4	Plasmid for the expression of AD-Rrm4mMLLE. Like AD-Rrm4, but carrying the amino acid substitutions F740A, I743A, A751G and K753A in the MLLE domain.	
pGBKT7-Upa1-Gfp 
	1785	upa1
	Plasmid for the expression of BD-Upa1-Gfp, where eGfp is fused C-terminally to the BD-Upa1-hybrid.	
pGBKT7-Upa1DN1-Gfp 	1964	upa1	Plasmid for the expression of BD-Upa1DN1-Gfp. Like BD-Upa1-Gfp, but carrying a N-terminal truncation from aa 1-143 including the PAM2-motif.	
pGBKT7-Upa1DN2-Gfp	1965	upa1	Plasmid for the expression of BD-Upa1DN2-Gfp. Like BD-Upa1-Gfp, but carrying a N-terminal truncation from aa 1-357 including the PAM2 and PAM2L-motifs.	
pGBKT7-Upa1DN3-Gfp	2030	upa1	Plasmid for the expression of BD-Upa1DN3-Gfp. Like BD-Upa1-Gfp, but carrying a N-terminal truncation from aa 1-551.	
pGBKT7-Upa1DN4-Gfp	2031	upa1	Plasmid for the expression of BD-Upa1DN4-Gfp. Like BD-Upa1-Gfp, but carrying a N-terminal truncation from aa 1-633.	
pGBKT7-Upa1DN5-Gfp	2032	upa1	Plasmid for the expression of BD-Upa1DN5-Gfp. Like BD-Upa1-Gfp, but carrying a N-terminal truncation from aa 1-719. 	
pGBKT7-Upa1DN6-Gfp	1966	upa1	Plasmid for the expression of BD-Upa1DN6-Gfp. Like BD-Upa1-Gfp, but carrying n N-terminal truncation from aa 1-969.	
pGBKT7-Upa1DN7DFR-Gfp 	2162	upa1	Plasmid for the expression of BD-Upa1GDN7/DFR. Like BD-Upa1-Gfp, but carrying only a region of upa1 ORF comprising of aa 883-1047.	
pGBKT7-Upa1DF-Gfp 	1791	upa1	Plasmid for the expression of BD- Upa1DF-Gfp. Like BD-Upa1-Gfp, but a region from aa 814-1156 including the FYVE domain was removed.	
pGBKT7-Upa1DN7DFR/Mut1-Gfp
	2219	upa1	Plasmid for the expression of BD-Upa1DN7DFR/Mut1-Gfp. Like BD-Upa1DN7DFR-Gfp, but carrying a block mutation with the amino acid substitutions AASAAATAAS from aa 886-895.	
pGBKT7-Upa1DN7DFR/Mut2-Gfp
	2220	upa1	Plasmid for the expression of BD-Upa1DN7DFR/Mut2-Gfp. Like BD-Upa1DN7/DFR-Gfp, but carrying a block mutation with the amino acid substitutions AASAAATAAS from aa 896-905.	
pGBKT7-Upa1DN7DFR/Mut3-Gfp
	2221	upa1	Plasmid for the expression of BD-Upa1DN7DFR/Mut3-Gfp. Like BD-Upa1DN7/DFR-Gfp but carrying a block mutation with the amino acid substitutions AASAAATAAS from aa 908-917.	
pGBKT7-Upa1DN7DFR/Mut4-Gfp
	2222	upa1	Plasmid for the expression of BD-Upa1DN7DFR/Mut4-Gfp. Like BD-Upa1DN7/DFR-Gfp, but carrying a block mutation with the amino acid substitutions AASAAATAAS from aa 918-927.	
pGBKT7-Upa1DN7DFR/Mut5-Gfp
	2223	upa1	Plasmid for the expression of BD-Upa1DN7DFR/Mut5-Gfp. Like BD-Upa1DN7/DFR-Gfp, but carrying a block mutation with the amino acid substitutions AASAAATAAS from aa 928-937.	
pGBKT7-Upa1DN7DFR/Mut6-Gfp
	2224	upa1	Plasmid for the expression of BD-Upa1DN7DFR/Mut6-Gfp. Like BD-Upa1DN7/DFR-Gfp, but carrying a block mutation with the amino acid substitutions AASAAATAAS from aa 938-947.	
pGBKT7-Upa1DN7DFR/Mut7-Gfp
	2225	upa1	Plasmid for the expression of BD-Upa1DN7DFR/Mut7-Gfp. Like BD-Upa1DN7/DFR-Gfp, but carrying a block mutation with the amino acid substitutions AASAAATAAS from aa 949-958.	
pGBKT7-Upa1DN7DFR/Mut8-Gfp
	2226	upa1	Plasmid for the expression of BD-Upa1DN7DFR/Mut8-Gfp. Like BD-Upa1DN7/DFR-Gfp, but carrying a block mutation with the amino acid substitutions AASAAATAAS from aa 959-968.	
pGBKT7-Upa1DN7DFR/Mut9-Gfp
	2227	upa1	Plasmid for the expression of BD-Upa1DN7DFR/Mut9-Gfp. Like BD-Upa1DN7/DFR-Gfp, but carrying a block mutation with the amino acid substitutions AASAAATAAS from aa 973-982.	
pGBKT7-Upa1DN7DFR/Mut10-Gfp
	2228	upa1	Plasmid for the expression of BD-Upa1DN7DFR/Mut10-Gfp. Like BD-Upa1DN7/DFR-Gfp, but carrying a block mutation with the amino acid substitutions AASAAATAAS from aa 983-992.	
pGBKT7-Upa1DN7DFR/Mut11-Gfp
	2229	upa1	Plasmid for the expression of BD-Upa1DN7DFR/Mut11-Gfp. Like BD-Upa1DN7/DFR-Gfp, but carrying a block mutation with the amino acid substitutions AASAAATAAS from aa 996-1005.	
pGBKT7-Upa1GDN7DFR/Mut12
	2230	upa1	Plasmid for the expression of BD-Upa1DN7DFR/Mut12-Gfp. Like BD-Upa1DN7/DFR-Gfp, but carrying a block mutation with the amino acid substitutions AASAAATAAS from aa 1021-1030.	
pGBKT7-Upa1N2-Gfp	1939	upa1	Plasmid for the expression of BD-Upa1N2-Gfp. Like BD-Upa1-Gfp, but carrying a C-terminal truncation from aa 410-1287.	
pGBKT7-Upa1N2/mP-Gfp	1940	upa1	Plasmid for the expression of BD-Upa1N2/mP-Gfp. Like BD-Upa1N2-Gfp, but carrying a C-terminal truncation from aa 410-1287 and carrying the nucleic acid exchanges T394G, T395C, G396C, G406C, C407T, T415G, T416C, G420A, C421G and G423T, generating the amino acid substitutions L132A, A136S, F139A and P141A in the PAM2-motif.	
pGBKT7-Upa1N2/mPL-1-Gfp	2307	upa1	Plasmid for the expression of BD-Upa1GN2/mPLN. Like BD-Upa1N2-Gfp, but carrying a C-terminal truncation from aa 410-1287 carrying block mutations leading to the amino acid substitutions AASAAATAAS from aa 242-251 in the N-terminal PAM2L-motif (PAM2L-1)	
pGBKT7-Upa1N2/mDFVYP-Gfp	2586	upa1	Plasmid for the expression of BD-Upa1N2/mDFVYP-Gfp. Like BD-Upa1N2-Gfp, but carrying a C-terminal truncation from aa 410-1287 carrying block mutations leading to the amino acid substitutions AAAAA from aa 247-251 in the N-terminal PAM2L-motif (PAM2L-1)	
pGBKT7-Upa1N2/mF248A-Gfp	2588	upa1	Plasmid for the expression of BD-Upa1N2/mF248A-Gfp. Like BD-Upa1N2-Gfp, , but carrying carrying the nucleic acid exchanges T742G and T743C, generating the amino acid substitution F248A in the N-terminal PAM2L-motif (PAM2L-1)	
pGBKT7-Upa1DN7DFR/mEFIYP-Gfp	2597	upa1	Plasmid for the expression of BD-Upa1DN7DFR/mEFIYP-Gfp. Like BD-Upa1DN7DFR-Gfp, but carrying a block mutation leading to the amino acid substitutions AAAAA from aa 954-958 in the C-terminal PAM2L-motif (PAM2L-2).	
pGBKT7-Upa1DN7DFR/mF955A-Gfp	2601	upa1	Plasmid for the expression of BD-Upa1DN7DFR/mF955A-Gfp. Like BD-Upa1DN7DFR-Gfp, but carrying the nucleic acid exchanges T2863G and T2864C, which results in the amino acid substitution F955A in the C-terminal PAM2L-motif (PAM2L-2).	
