# Supplementary material for: A FYVE zinc finger domain protein specifically links mRNA transport to endosome trafficking
Source: eLife. 2015 May 18;4:e06041. doi: 10.7554/eLife.06041 (PMC4466420; doi:10.7554/eLife.06041)
Supplement: Supplementary file 6. — Description of plasmids used for pulldown experiments. DOI: http://dx.doi.org/10.7554/eLife.06041.052 [file elife06041s006.rtf]

Supplementary file 6: Description of plasmids used for pulldown experiments
Plasmid	pUMa	Short description	
pGEX_GST	1881	Plasmid for the expression of the GST (Glutathione S transferase)-tag alone. Expression is regulated by tac promoter. The plasmid also contains a lacIq gene for use in E. coli. The plasmid carries an ampicillin resistance for selection. This vector is based on pGEX-2T (GE Healthcare).	
pGEX_GST-MLLEPab1	2187	Plasmid for the expression of the GST-MLLEPab1. The last 86 aa of the pab1 ORF including the MLLE domain were N-terminally fused to a GST-tag.	
pGEX_GST-Rrm4DN5	2385	Plasmid for the expression of the GST-Rrm4DN5. A region of Rrm4 comprising of amino acid 720-792 was N-terminally fused to a GST-tag.	
pET15B_Upa1N2	2380	Plasmid for the expression of a His6-Upa1N2 in E. coli. N-terminal fragment of Upa1 (aa 1-363) carrying a N-terminal fusion of a 6x histidine-tag. Expression is regulated by lacO. The plasmid carries an ampicillin resistance for selection.	
pET15B_Upa1N2mPAM2	2381	Plasmid for the expression of a His6-Upa1N2 in E. coli. Like pET15B_Upa1N2, but carrying the nucleic acid exchanges T394G, T395C, G396C, G406C, C407T, T415G, T416C, G420A, C421G and G423T generating the amino acid substitutions L132A, A136S, F139A and P141A in the PAM2-motif.	
pET15B_Upa1N2mPAM2L	2382	Plasmid for the expression of a His6-Upa1N2 in E. coli. Like pET15B_Upa1N2, but carrying block mutations leading to the amino acid substitutions AASAAATAAS from aa 949-958.	
