# Supplementary material for: A FYVE zinc finger domain protein specifically links mRNA transport to endosome trafficking
Source: eLife. 2015 May 18;4:e06041. doi: 10.7554/eLife.06041 (PMC4466420; doi:10.7554/eLife.06041)
Supplement: Supplementary file 7. — DNA oligonucleotides used in this study. DOI: http://dx.doi.org/10.7554/eLife.06041.053 [file elife06041s007.rtf]

Supplementary file 7: DNA oligonucleotides used in this study 
Designation	Nucleotide sequence (5' --> 3')	Remarks	
oMF503	TGTGGCCGTTTACGTCGC		
oMF369	TTCACACAGGAAACAGCTATGACC		
oMF963	TGGAAAGCGGGCAGTGAG		
oSL99	AGCGCAACAACGCCGATG		
oRL305	AATATTTGCACAAATGAACGGCTCC	upa1 u2 	
oRL306	AATATTCCTGTTTTGCTATACCCTTAGCG	upa1 d2 	
oRL307	TTGGGCCATCTAGGCCGTCGATTCAGACCATGCAAG	upa1 u3 UF 	
oRL308	AATGGCCGCGTTGGCCGCCCAATCTCTGGCGTGCACAG	upa1 u3 UF + ORF	
oRL309	TGAGGCCTGAGTGGCCATGGTCCCATACGGGTTTCG	upa1 d1	
oRL310	ACGACTAGGTGACGTCCG	upa1 d3	
oRL311	CGCACTGCAATCGCACTG	upa1 u1	
oRL312	GCTCTCTGCCCTACAAGC	upa1 p1	
oRL313	GGCTGAGACGACCACTTG	upa1 p2	
oRL323	CAAGTGGCTCGTAGGGAC		
oRL340	AATGGCCGCGTTGGCCGCGTTCTGCTCGCGCATGGTG	upa1DFR	
oRL392	GCACTGCACGCCGTAGG		
oRL395	AATGGCCGCGTTGGCCGCGCCCACGATTGGCGAGTC	upa1DR	
oRL423	TGAGGCCATTACGGCCCATATGTCCGATTCGATTTACGCC	rrm4 for Y2H	
oRL424	CGTGGCCGAGGCGGCCGTCACTTGTTCAGACCTGCAGC	rrm4 for Y2H	
oRL594	TGAGGCCATTACGGCCCATATGTCGTCCACCGAGTCC	pab1 for Y2H	
oRL595	CGTGGCCGAGGCGGCCGTCAAGCCTCAGTCTTGGGAGC	pab1 for Y2H	
oRL596	TGAGGCCATTACGGCCCATATGACGATACCAGACCCG	upa1 for Y2H	
oRL597	CGTGGCCGAGGCGGCCGTCACCAATCTCTGGCGTGCAC	upa1 for Y2H	
oRL671	GGTAAGGGCCCCGGCAAGCTCGGGCTGGCTGGCGGCAGCCTTGGGGGCGATGGCCTCACC	mMLLEPab1	
oRL672	TTGCCGGGGCCCTTACCGGTATGGCCCTTGAGCTT	mMLLEPab1	
oRL754	GGGAAGCTTTGGCGACGGAGAGGTTGGGAGAGGCGGTGCTTTGCG	upa1mP	
oRL755	CTTCAAAGCTTCCCGATCCTTGCAACCG	upa1mP	
oRL1103	TACGGCCATTACGGCCAGATCTAGTACTATGTCCTTGCAACCGAATCAC	upa1DN1	
oRL1104	TACGGCCATTACGGCCAGATCTTCTAGAATGCTCTCCAACGACCCAAAC	upa1DN2	
oRL1105	TACGGCCATTACGGCCAGATCTACTAGTATGACTCGTCTGGTCAACGGC	upa1DN6	
oRL1106	GTCAGATCTGTCGATTCAGACCATGCAAG		
oRL1115	CGTGACCGCGGCCT	SfiI-SfiI-Linker	
oRL1116	CCGCGGTCACGCGT	SfiI-SfiI-Linker	
oRL1121	TACGGCCATTACGGCCAGATCTGATATCATGCCCTACGAGCCACTTGCC	upa1DN3	
oRL1122	TACGGCCATTACGGCCAGATCTGATATCATGTCTCTGCCCTACAAGCTG	upa1DN4	
oRL1123	TACGGCCATTACGGCCAGATCTCATATGGTAGACGTCGAACACGGC	upa1DN5	
oRL1217	TGAGGCCATTACGGCCCATGCGACGGTCAAGGCAACC	MLLERrm4 for Y2H	
oRL1426	TACGGCCATTACGGCCTCTAGAGATATCATGCCTCACCTCACTCGCCCC	rrm4DN1	
oRL1427	TACGGCCATTACGGCCTCTAGAACTAGTATGGCCAAACCACGTCTTGTC	rrm4DN2	
oRL1428	TACGGCCATTACGGCCTCTAGACGATCGATGAGTCCGTCAGCTCCG	rrm4DN3	
oRL1429	TACGGCCATTACGGCCTCTAGATGTACAATGAGCAGCAACAGTCCG	rrm4DN4	
oRL1514	TCAGGTCTCGCCTGCGCTGACTAGGGCACATC	Mut u1	
oRL1515	TCAGGTCTCGCTGCCGCCCGGAATTAGCTTGG	Mut u2	
oRL1516	GGTCTCGCGGCCACCGCCGCCTCGCGTCTCGTCATGGCCAATAC	Mut1 p1	
oRL1517	GGTCTCGGCCGCGGCCGAGGCGGCAGCGCCCATAGTACTAGATC	Mut1 p2	
oRL1518	GGTCTCGCGGCCACCGCCGCCTCGGCCGCTTTCGAAGACGATG	Mut2 p1	
oRL1519	GGTCTCGGCCGCGGCCGAGGCGGCAGCGATACGCCTGGCGAG	Mut2 p2	
oRL1520	GGTCTCGCGGCCACCGCCGCCTCGCGCGCTGAAGAGCTGCGAAAG	Mut3 p1	
oRL1521	GGTCTCGGCCGCGGCCGAGGCGGCAGCGGCGCGCAGCTCTGTATTG	Mut3 p2	
oRL1522	GGTCTCGCGGCCACCGCCGCCTCGCTGCAGCTCGGACAACTG	Mut4 p1	
oRL1523	GGTCTCGGCCGCGGCCGAGGCGGCGCTTCTACGCTCGACGTC	Mut4 p2	
oRL1524	GGTCTCGCGGCCACCGCCGCCTCGGGCTTGGACGAAGACGAG	Mut5 p1	
oRL1525	GGTCTCGGCCGCGGCCGAGGCGGCCGCGTACTCCTTTCGCAG	Mut5 p2	
oRL1526	GGTCTCGCGGCCACCGCCGCCTCGGCCGATGACGATGACGATG	Mut6 p1	
oRL1527	GGTCTCGGCCGCGGCCGAGGCGGCGGCGGCCTCGCGCAGTTG	Mut6 p2	
oRL1528	GGTCTCGCGGCCACCGCCGCCTCGAACTCGTACAAGGACGATGG	Mut7 p1	
oRL1529	GGTCTCGGCCGCGGCCGAGGCGGCGGCCGCATCCTCGTCCTC	Mut7 p2	
oRL1530	GGTCTCGCGGCCACCGCCGCCTCGTTCACTCGTCTGGTCAACGG	Mut8 p1	
oRL1531	GGTCTCGGCCGCGGCCGAGGCGGCCGGGTAGATGAATTCATCGTC	Mut8 p2	
oRL1532	GGTCTCGCGGCCACCGCCGCCTCGCGACCCTCACTCTCTCAG	Mut9 p1	
oRL1533	GGTCTCGGCCGCGGCCGAGGCGGCCAGACGAGTGAAGACAGAC	Mut9 p2	
oRL1534	GGTCTCGCGGCCACCGCCGCCTCGGCCGCCTCGATGCTGCGC	Mut10 p1	
oRL1535	GGTCTCGGCCGCGGCCGAGGCGGCAGAGAGGGTGCCTGAAGTC	Mut10 p2	
oRL1536	GGTCTCGCGGCCACCGCCGCCTCGGCCGGTACGTCGCTCGCTG	Mut11 p1	
oRL1537	GGTCTCGGCCGCGGCCGAGGCGGCCGAGGCGGCACTACTCTG	Mut11 p2	
oRL1538	GGTCTCGCGGCCACCGCCGCCTCGGCGCCAAATAAGGTTGTGC	Mut12 p1	
oRL1539	GGTCTCGGCCGCGGCCGAGGCGGCGGCGGCGAGGTCAGCGCTG	Mut12 p2	
oRL1827	TACGCGGCCGCCGAGGCGGCTTGGTCTGCAGCCTCAGTG	bmPAM2L-1	
oRL1828	TACGCGGCCGCCACCGCCGCCTCGGGAGCCGACAGCCCAAGTAG	bmPAM2L-1	
oRL1918	TACGGCCATTACGGCCTCTAGAGATATCATGCCCAAGGTTTCCTCGGTC	rrm4DN5	
oRL1919	TACGGCCATTACGGCCTCTAGAACTAGTATGCTCGGTATTCAGGCTC	rrm4DN6	
oRL1920	TACGGCCATTACGGCCTCTAGACGATCGATGCCGGTGCCATTGTCAGTG	rrm4DN7	
oDD442	ATTGCTCTTCCGTGCGCTGACTAGGGCACATC	mPAM2L-1	
oDD443	ATTGCTCTTCCCGGCCGCGGCATCTTGATCTTCCTCTTGGTC	mDFVYP	
oDD444	ATTGCTCTTCGCCGCCGCAGGAGCCGACAGCCC	mDFVYP	
oDD445	ATTGCTCTTCCGACAGACCCGTTTAGAGGCCC	mPAM2L-1	
oDD450	ATTGCTCTTCCTACCCGGGAGCCGACAGCC	mF248A	
oDD451	ATTGCTCTTCCGTAGACAGCGTCATCTTGATCTTCCTCTTGG	mF248A	
oDD470	GGTCTCCCGGCCGCTGCATCGTCATCGTCATCGGCCG	mEFIYP	
oDD471	GGTCTCCGCCGCCGCGAACTCGTACAAGGACGATG	mEFIYP	
oDD472	GGTCTCCAGATGGCTTCATCGTCATCGTCATCGG	mF955A	
oDD473	GGTCTCCATCTACCCGAACTCGTACAAG	mF955A	
